# Management of Catastrophic Postpartum Acute Pulmonary Embolism With Extracorporeal Membrane Oxygenation Support and Thromboaspiration: A Case Report

**DOI:** 10.1155/crog/5555153

**Published:** 2026-09-09

**Authors:** Fabio Varón-Vega, María Camila Martínez-Ayala, Eduardo Tuta-Quintero, Dario Echeverri, José Rojas-Suarez

**Affiliations:** ^1^ Critical Care Service, Fundación Cardioinfantil-Instituto de Cardiologia, Bogotá, Colombia; ^2^ Critical Care and Lung Transplantation Service, Fundación Neumológica Colombiana, Bogotá, Colombia; ^3^ School of Medicine, Universidad de la Sabana, Chia, Colombia, unisabana.edu.co; ^4^ Interventional Cardiology Lab, Fundación Cardioinfantil-Instituto de Cardiologia, Bogotá, Colombia; ^5^ Intensive Care Medicine and Obstetric Research Group (GRICIO), Universidad de Cartagena, and GINUMED Group, Corporación Universitaria Rafael Núñez, Cartagena, Colombia, curn.edu.co

**Keywords:** case report, critical care, extracorporeal membrane oxygenation, postpartum period, pulmonary embolism

## Abstract

**Background:**

Pulmonary embolism (PE) is a leading cause of maternal mortality during the postpartum period and is associated with significant hemodynamic instability. High‐risk PE, characterized by obstructive shock and right ventricular failure, requires immediate and aggressive intervention. In cases refractory to conventional therapies, extracorporeal membrane oxygenation (ECMO) may serve as a life‐saving bridge to definitive treatment.

**Case Description:**

We report the case of a 28‐year‐old woman who developed high‐risk acute PE 48 h after an uncomplicated vaginal delivery. She presented with severe obstructive shock and right ventricular failure unresponsive to systemic thrombolysis and standard hemodynamic support. Due to progressive clinical deterioration, venoarterial ECMO was initiated as a rescue therapy, which led to hemodynamic stabilization. Mechanical thromboaspiration was subsequently performed, achieving successful thrombus removal and clinical improvement.

**Conclusion:**

This case highlights the critical role of ECMO as a therapeutic bridge in patients with high‐risk PE and hemodynamic collapse. The combination of ECMO with catheter‐directed thromboaspiration proved effective in restoring hemodynamic stability and resolving thrombotic obstruction, offering a viable rescue strategy in life‐threatening postpartum PE.

## 1. Introduction

Acute pulmonary embolism (PE) is a serious and potentially fatal complication in the postpartum period, with an estimated incidence of approximately 1.72 per 1000 births, making it one of the leading causes of maternal morbidity and mortality worldwide [1, 2]. High‐risk PE in this context can present dramatically, often with hemodynamic instability, severe hypoxia, and cardiovascular collapse, necessitating prompt and effective intervention to prevent fatal outcomes [3, 4]. The management of such patients is particularly challenging due to the physiological changes associated with pregnancy and the postpartum state, which predispose to a hypercoagulable condition and increase the risk of thrombotic events [4].

In cases of high‐risk PE with obstructive shock, extracorporeal membrane oxygenation (ECMO) has emerged as a key therapeutic tool to provide temporary cardiopulmonary support, enabling hemodynamic stabilization while other treatment strategies, such as thrombolysis or mechanical thromboaspiration, are implemented [5, 6]. ECMO provides extracorporeal oxygenation and circulatory support, reducing right ventricular overload and facilitating cardiovascular recovery [6]. Thromboaspiration has proven effective as a mechanical strategy for thrombus removal, quickly restoring pulmonary blood flow and improving gas exchange [7]. The combination of ECMO and thromboaspiration has shown promising results in patients with high‐risk PE refractory to conventional treatments [5–7].

However, the application of this combined approach in postpartum patients is rarely described in the literature and presents specific challenges. These include the risk of hemorrhage secondary to required systemic anticoagulation for circuit patency, alongside potential complications such as coagulopathy, infection, and multiorgan dysfunction [8]. The decision to initiate ECMO in this setting requires careful evaluation of risks and benefits, considering the patient′s clinical stability, contraindications to anticoagulation, and the feasibility of successful thromboaspiration.

The objective of this report is to present the successful management of a case of postpartum high‐risk PE using combined ECMO and thromboaspiration, emphasizing the importance of early identification and a multidisciplinary approach to achieve a favorable outcome. This case highlights the role of ECMO as a rescue therapy in critical situations and provides valuable insights to inform future clinical decision‐making in similar scenarios.

## 2. Case Report

We present the case of a 28‐year‐old woman (weight: 65 kg, height: 165 cm, body mass index: 23.8 kg/m^2^), with no known prior medical history, at 38.4 weeks of gestation (G2V2P2), admitted for labor induction at an outside facility for a routine term delivery. The vaginal delivery proceeded without complications, and the female newborn was healthy. At 24 h postpartum, the patient underwent bilateral tubal ligation (Pomeroy procedure) via a laparoscopic approach under general anesthesia, without intraoperative complications. Postoperative thromboprophylaxis with thigh‐high, medium antiembolism elastic compression stocking was ordered and placed; pharmacological prophylaxis was deferred to prevent early postsurgical bleeding.

In the immediate postoperative period, the patient experienced a syncopal episode followed by cardiopulmonary arrest (asystole and ventricular tachycardia), requiring advanced cardiopulmonary resuscitation for 4 min and two defibrillations. A diagnosis of acute PE was confirmed via chest CT angiography and transthoracic echocardiography, which revealed right ventricular dysfunction with severe hemodynamic instability, necessitating vasopressor support with norepinephrine, vasopressin, and continuous epinephrine infusion. Systemic thrombolysis with alteplase 100 mg was administered due to persistent obstructive shock and imminent risk of death. Unfractionated heparin was considered and initiated but proved insufficient to resolve the acute mechanical obstruction. Ongoing instability and localized bleeding prompted transfer to our facility for venoarterial (VA) ECMO and mechanical thrombectomy.

Upon admission, the patient had borderline mean arterial pressure despite triple vasopressor support and exhibited active vaginal and airway bleeding. Point‐of‐care ultrasound (POCUS) revealed right ventricular dilation, a tricuspid annular plane systolic excursion (TAPSE) of 12 mm, and moderate tricuspid regurgitation. Physical examination of the lower extremities was symmetric without unilateral swelling or signs of deep vein thrombosis (DVT). A subsequent lower limb venous Doppler ultrasound did not identify any primary venous thrombotic focus, suggesting that the thromboembolism likely originated from the pelvic/uterine veins. Peripheral VA ECMO was initiated via femoral–femoral access, with a 19‐Fr arterial cannula in the right femoral artery and a reperfusion cannula, along with a 25‐Fr venous cannula in the left femoral vein. Precannulation arterial blood gases showed hypercapnia, hyperlactatemia, and anemia, necessitating transfusion (Table 1).

**Table 1:** Evolution of biochemical and arterial blood gas parameters.

Parameter	Admission	Post‐ECMO	Postthrombectomy	Postdecannulation
pH	7.0	7.26	7.42	7.27
pCO_2_ (mmHg)	82	49	33	41
Lactate	1.8	1.1	1.3	0.8
Hemoglobin (g/dL)	9.3	9.2	8.1	8.8
Creatinine (mg/dL)	3.1	3.3	5.6	4.0

**Figure 1:**
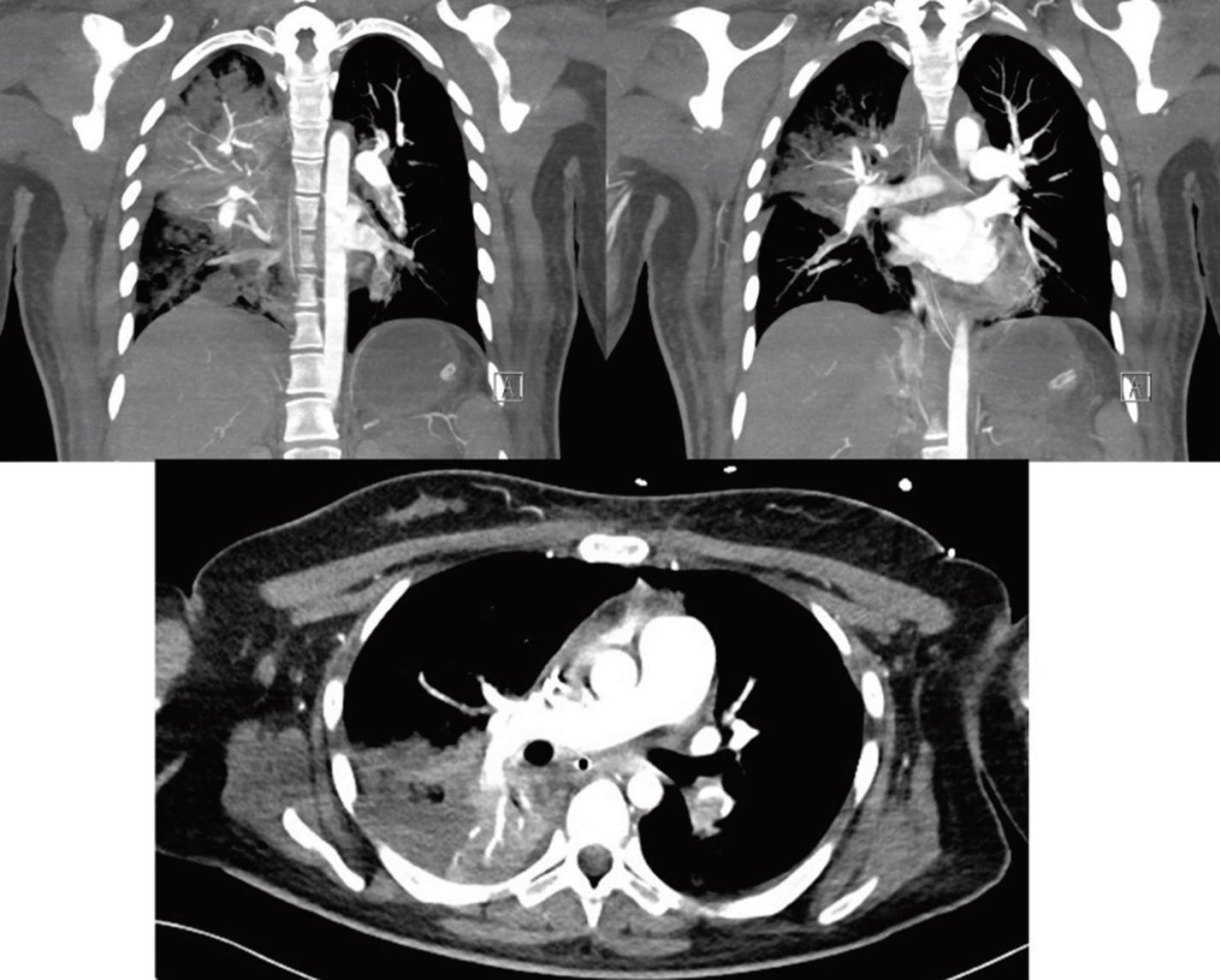
Admission chest CT angiography.

**Figure 2:**
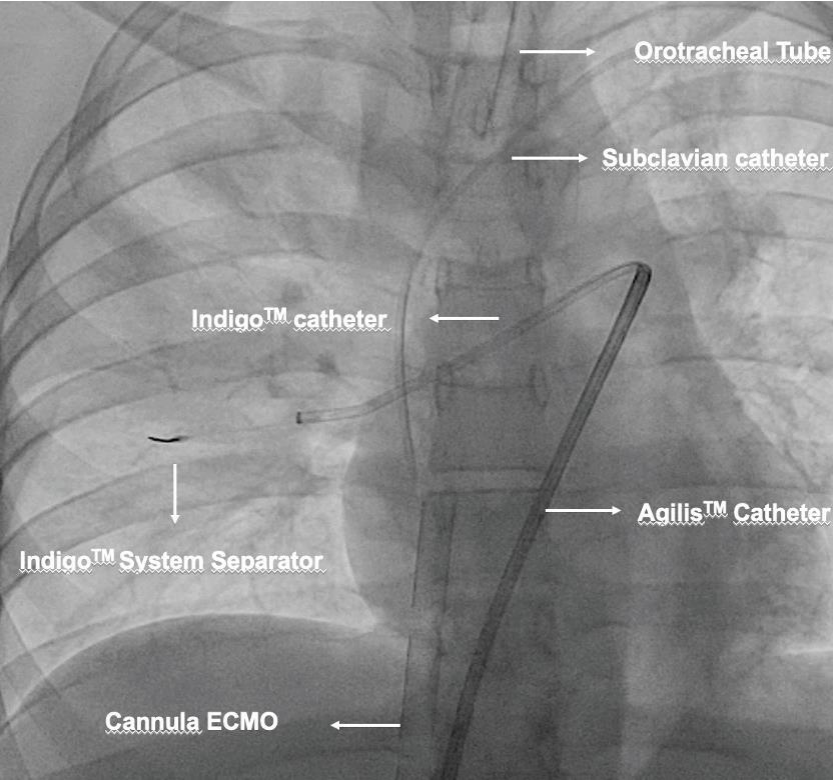
Penumbra Indigo catheter in the pulmonary artery.

Institutional CT angiography confirmed multiple pulmonary artery thrombi and signs of precapillary pulmonary hypertension (Figure 1). During VA‐ECMO cannulation an initial intravenous bolus of 6000 UI of unfractionated heparin was administered, achieving an initial activated clotting time (ACT) of 349 s, which settled at 210 s postprocedure. Continuous systemic anticoagulation was maintained using unfractionated heparin starting at 12 UI/kg/h and titrated according to an institutional nomogram, targeting an activated partial thromboplastin time (aPTT) of 50–70 s. Due to localized bleeding at the cannulation sites and active vaginal hemorrhage, the infusion was temporarily suspended and later resumed at a dose of 15 UI/kg/h maintaining an aPTT of 68 s. Pulmonary angiography via right femoral venous access showed extensive proximal and distal thrombotic burden with generalized distal hypoperfusion, involving Segments A1, A5–A10 on the right and A5, A7–A10 on the left. Hemodynamic measurements showed pulmonary artery pressures of 44/23/32 mmHg and right ventricular pressures of 43/8/15 mmHg, consistent with severe precapillary pulmonary hypertension due to thromboembolic disease (Figure 1). Mechanical pulmonary thromboaspiration was subsequently performed via the right femoral venous access under fluoroscopic guidance a deflectable Agilis NxT catheter (Abbott) was navigated through the inferior vena cava, right atrium, tricuspid valve, and right ventricle into the pulmonary artery trunk. Over this, an 8‐Fr Indigo Thrombectomy System (Penumbra, Inc., Alameda, California) was deployed. Multiple aspiration passes were made in both pulmonary arteries, directly targeting thrombi in the main trunk as well as segmental and subsegmental branches, specifically Segments A1, A5–A10 on the right and A5, A7–A10 on the left (Figure 2). Unfraction heparin infusion was briefly held during the active aspiration phase of the procedure to minimize bleeding risk.

Following the procedure, the patient showed favorable clinical progression, with gradual weaning of vasopressors until full discontinuation. The procedure achieved partial restoration of flow and reduction of thrombotic burden, contributing to progressive hemodynamic stabilization in conjunction with ongoing ECMO support. Renal function progressively improved, with complete recovery from KDIGO Stage 3 acute kidney injury, characterized by a rise in serum creatinine from 3.1 mg/dL on admission to a peak of 5.6 mg/dL and transient anuria. The acute kidney injury was attributed to severe obstructive and hypovolemic shock and resolved without the need for renal replacement therapy.

Follow‐up echocardiography performed 2 days later revealed a left ventricular ejection fraction (LVEF) of 45%–50%, mild to moderate tricuspid regurgitation, and an estimated pulmonary artery systolic pressure (PASP) of 41 mmHg. Compared with baseline, there was improved right ventricular function, reduced chamber dilation (prior PASP 51 mmHg, TAPSE 24 mm), and resolution of left ventricular hyperkinesis.

ECMO decannulation was performed on Day 3, following resolution of tachycardia, improved perfusion, reduced hyperdynamic signs, and stable hemodynamics. The patient required low‐dose dobutamine, which was tapered and discontinued. She was weaned off invasive mechanical ventilation on Day 5 and continued anticoagulation with unfractionated heparin without complications, progressing to standard inpatient care.

**Figure 3:**
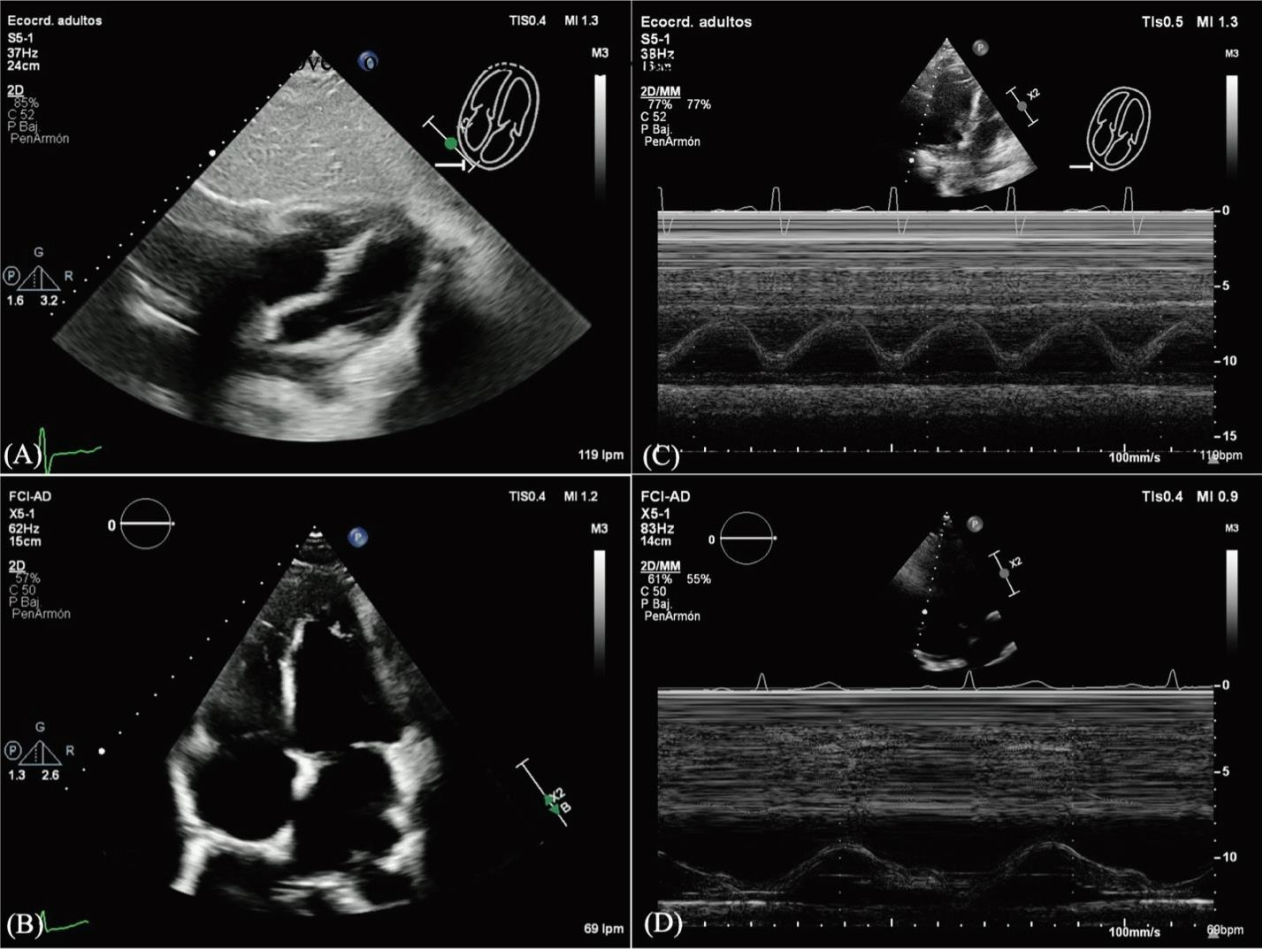
Comparative echocardiographic findings between admission and 3‐month follow‐up. (A) Apical four‐chamber view on admission showing right ventricular dilation and septal flattening consistent with pressure overload. (B) Follow‐up apical four‐chamber view showing normalization of right ventricular size and septal position. (C) TAPSE measurement at admission showing reduced longitudinal right ventricular function (12 mm). (D) Follow‐up TAPSE demonstrating recovery of right ventricular systolic function (27.7 mm).

At 3‐month follow‐up, the patient remained in good clinical condition—fully functional, asymptomatic, and without limitations for daily activities. Follow‐up transthoracic echocardiography demonstrated normalization of biventricular function. The left ventricle showed normal size and wall thickness, no regional wall motion abnormalities, normal diastolic function, and a preserved ejection fraction of 63%. The right ventricle also appeared normal, with preserved systolic function. No residual pulmonary hypertension was noted, with an estimated PASP within normal limits. Comparative echocardiographic findings between admission and follow‐up, including recovery of right ventricular size and function as reflected by TAPSE, are shown in Figure 3.

## 3. Discussion

High‐risk PE in the postpartum period remains one of the foremost causes of maternal death, frequently presenting with hemodynamic collapse, hypoxia, and cardiovascular failure [3, 4]. In this case, the combined use of ECMO and thromboaspiration achieved hemodynamic stabilization and rapid thrombus resolution, enabling favorable clinical recovery. A multidisciplinary approach was essential to reversing obstructive shock and improving maternal prognosis, underscoring ECMO′s vital role as a rescue therapy in patients unresponsive to conventional treatments.

Numerous studies have documented ECMO′s effectiveness in high‐risk PE with obstructive shock [9]. For instance, a systematic review by Scott et al. [10] reported survival rates up to 61% in patients with cardiac arrest secondary to massive PE managed with VA‐ECMO. Similarly, Kmiec et al. [11] found that patients receiving additional therapies, such as surgical thrombectomy post‐ECMO stabilization, had survival outcomes comparable with those treated with anticoagulation alone, supporting ECMO as a bridge‐to‐therapy in high‐risk PE.

Implementing ECMO in the postpartum context involves unique challenges. Systemic anticoagulation, necessary to prevent ECMO circuit thrombosis, increases the risk of postpartum hemorrhage and obstetric complications [12]. In our patient, the PE occurred against the physiological hypercoagulable backdrop of the early postpartum period, compounded by the surgical stress, pneumoperitoneum, and transient immobilization of the laparoscopic Pomeroy procedure. Systemic thrombolysis (alteplase 100 mg) was selected as a life‐saving rescue therapy because of refractory cardiac arrest and obstructive shock, which outweighed the high risk of postpartum hemorrhage. Unfrationated heparin was considered and initiated initially but proved insufficient to restore perfusion. A formal thrombophilia panel was deferred during the acute phase due to ongoing anticoagulation and is scheduled for outpatient follow‐up. Favorable outcomes, as in our case, have been reported in similar peripartum scenarios, such as those described by Park et al. [13] and Leeper et al. [14], where ECMO provided hemodynamic support and facilitated recovery in critically ill postpartum patients.

The synergistic combination of ECMO and thromboaspiration, as illustrated in this case, appears to enhance hemodynamic stabilization. This case reinforces the viability and efficacy of this combined strategy in postpartum patients, even when hemorrhagic risks are elevated. Different types of catheters are used for mechanical fragmentation, thrombus aspiration, or more commonly, a pharmacochemical approach combining mechanical or ultrasound fragmentation of the thrombus with in situ reduced‐dose thrombolysis. The Indigo Thrombectomy System (Penumbra, Inc., Alameda, California) is a smaller bore aspiration catheter designed to engage thrombus and extract it with a continuous vacuum pump; it has been cleared by both the FDA and CE Mark, supporting its safety and efficacy in mechanical thrombectomy procedures.

This report highlights the critical importance of timely intervention and multidisciplinary coordination in the management of postpartum PE. ECMO offers essential hemodynamic and respiratory support, whereas thromboaspiration enables rapid thrombus resolution, resulting in swift clinical improvement. Accumulating clinical experience and recent studies suggest that this combined approach could evolve into a reference standard for managing high‐risk PE in postpartum patients. Nonetheless, further research is necessary to develop standardized protocols and identify patient profiles that would benefit most from this combined therapy.

## 4. Conclusion

The successful management of postpartum high‐risk PE using ECMO and thromboaspiration highlights the importance of a comprehensive and multidisciplinary therapeutic strategy in hemodynamically unstable patients. ECMO provided essential circulatory and respiratory support, whereas thromboaspiration facilitated rapid thrombus resolution, enabling right ventricular recovery and hemodynamic stabilization. This case reinforces existing evidence supporting ECMO as a rescue therapy in refractory high‐risk PE. The experience derived from this case contributes to the growing body of knowledge on high‐risk PE management and highlights the need for continued investigation and standardized protocols to optimize treatment and improve survival in this high‐risk population.

## Funding

No funding was received for this manuscript.

## Ethics Statement

Written informed consent was obtained from the individual for the publication of any potentially identifiable images or data included in this article. Written informed consent has been obtained from the participant for the publication of this case report.

## Conflicts of Interest

The authors declare no conflicts of interest.

## Data Availability

The original contributions presented in the study are included in the article/supporting material; further inquiries can be directed to the corresponding author.
